# CIC reduces xCT/SLC7A11 expression and glutamate release in glioma

**DOI:** 10.1186/s40478-023-01507-y

**Published:** 2023-01-16

**Authors:** Jong-Whi Park, Omer Kilic, Minh Deo, Kevin Jimenez-Cowell, Engin Demirdizen, Hyunggee Kim, Şevin Turcan

**Affiliations:** 1 Neurology Clinic and National Center for Tumor Diseases, University Hospital Heidelberg, INF 460, 69120 Heidelberg, Germany; 2 Department of Life Sciences, Gachon University, Incheon, 21999 South Korea; 3 Department of Biotechnology, College of Life Sciences and Biotechnology, Korea University, Seoul, 02841 South Korea

**Keywords:** Oligodendroglioma, Capicua, Glutamate, Neuronal toxicity, xCT/SLC7A11

## Abstract

Capicua (CIC) is an important downstream molecule of RTK/RAS/MAPK pathway. The regulatory mechanism of CIC underlying tumorigenesis in oligodendroglioma, where CIC is frequently mutated, has yet to be fully elucidated. Using patient-derived glioma lines, RNA-sequencing and bioinformatic analysis of publicly available databases, we investigated how CIC loss- or gain-of-function regulates its downstream targets, cell proliferation and glutamate release. Our results indicate an increased frequency of CIC truncating mutations in oligodendroglioma during progression. In vitro, CIC modulation had a modest effect on cell proliferation in glioma lines, and no significant changes in the expression of *ETV1, ETV4* and *ETV5*. Transcriptional repression of known CIC targets was observed in gliomas expressing non-phosphorylatable CIC variant on Ser173 which was unable to interact with 14-3-3. These data outline a mechanism by which the repressor function of CIC is inhibited by 14-3-3 in gliomas. Using transcriptional profiling, we found that genes related to glutamate release were upregulated because of CIC depletion. In addition, loss of CIC leads to increased extracellular glutamate. Consistent with this, CIC restoration in an oligodendroglioma line reduced the levels of extracellular glutamate, neuronal toxicity and xCT/SLC7A11 expression. Our findings may provide a molecular basis for the prevention of glioma-associated seizures.

**Supplementary Information:**

The online version contains supplementary material available at 10.1186/s40478-023-01507-y.

## Introduction

Isocitrate dehydrogenase mutation (IDH) and co-deletion of chromosome arms 1p and 19q (codel) are diagnostic features of oligodendrogliomas (ODGs) [19]. ODGs are further characterized by somatic alterations in *CIC* and *FUBP1* on the residual alleles of 1p/19q [2]. Subsequent studies demonstrated that CIC mutations occur in approximately 50–70% of ODG [8, 36], with poor survival outcomes [8, 12].

At the molecular level, CIC is a conserved transcriptional repressor downstream of the receptor tyrosine kinase (RTK) and mitogen-activated protein kinase (MAPK) pathways [14]. RTK-mediated MAPK activation phosphorylates and mitigates the repressor activity of CIC via its cytoplasmic export from the nucleus or degradation [15, 29]. Post-transcriptional regulation of CIC has been shown in glioblastoma (GBM) [5]. Importantly, phosphorylation on serine 173 (S173) residue plays a crucial role in ubiquitin-mediated degradation of CIC. The phosphorylated S173 has also been reported to bind to 14-3-3 proteins, resulting in reduced DNA binding activity of CIC [10].

Structurally, CIC contains the high mobility group (HMG)-box and the C1 domain, recognizing T(G/C)AATG(A/G)A sequence for the repression of target genes [11]. In ODG, CIC mutations are frequently detected in the above two conserved domains, leading to higher expression of the oncogenic ETS/Pea3 transcription factors, including ETV1, ETV4 and ETV5 [22]. In this study, we investigated whether CIC restoration limits the oncogenic properties in ODG.

## Materials and methods

### Cell lines and cell culture

TS603, TS667, TS543 were obtained from Memorial Sloan Kettering Cancer Center (MSKCC). SU-AO3, NCH612 glioma lines were described previously [23]. The patient-derived IDH1-mutant glioma lines were routinely subjected to panel sequencing analysis [26] and immunoblotting to confirm the expression of mutant IDH1 (R132H). Glioma lines were maintained as neurospheres in NeuroCult (StemCell Technologies) with 2 µg/ml heparin sulfate (Sigma), 20 ng/ml of EGF (StemCell Technologies), and FGF2 (StemCell Technologies). PC12 cells (provided by Dr. Agarwal, Heidelberg University; Dr. Chang, Gachon University) were maintained in DMEM (D5796, Sigma) supplemented with 10% horse serum (H1138, Sigma) and 1% penicillin–streptomycin. Human neural progenitor cells (SCC008, Sigma) were maintained in DMEM (D5796, Sigma) supplemented with 10% fetal bovine serum (Gibco) and 1% penicillin–streptomycin. All cells were grown in a 5% CO_2_ humidified incubator at 37 °C.

### Plasmids and reagents

pCMV5 HA Capicua was purchased from MRC-PPU. For stable CIC expression, the CIC cDNA was subcloned into lentiviral vector (Flag-tagged) using Gateway system. CIC S173A mutant was generated using site-directed mutagenesis. Two independent oligos targeting CIC (sgCIC1: GCTCAGACACCAAGGCTCCG, sgCIC2: CCCCTCCGTGCAGCCGAGCG) were constructed and ligated into lentiCRISPR v2 (Addgene plasmid #52,961) for CIC depletion. pLenti CMV-SLC7A11-sh926R-FLAG-IRES-Hygro (Addgene plasmid #118,702) was used for xCT overexpression. The following reagents were used for in vitro treatments: monosodium glutamate (G1626, Sigma), R18 (2144, TOCRIS) and ( +)-MK801 maleate (HB0004, Hellobio).

### Immunostaining and Immunoblotting

5000 cells were seeded in laminin-coated 24-well plates. After fixation with 4% paraformaldehyde, cells were permeabilized with 0.1% Triton X100 for 15 min at room temperature. The cells were then incubated overnight at 4 ℃ with CIC antibody (NB110-59,906, NOVUS). Samples were rinsed three times in PBS and incubated for 1 h at room temperature with AlexaFluor 594 donkey anti-rabbit secondary antibody (Thermo Fisher Scientific). Nuclei were stained with 0.5 µg/ml DAPI for 1 min at room temperature. Images were acquired using fluorescence microscopy. The primary antibodies used for the immunoblots: anti-CIC (NB110-59,906, NOVUS), anti-ETV4 (10,684–1-AP, Proteintech), anti-xCT (NB300-318, NOVUS), anti-ACTB (A3854, Sigma), anti-GAPDH (2118, Cell Signaling).

### Soft-agar anchorage-independent growth measurement

To measure cellular anchorage-independent growth, 100,000 cells were seeded between 0.4% (top) and 0.8% (bottom) agar (Lonza). After two weeks, the cells were then stained with 0.005% Crystal Violet and imaged using Bio-Rad ChemiDoc MP imaging system. For cell proliferation measurement, cells were seeded at 200,000 cells per well in 6-well plates, and counted using a hemocytometer 4 days later. The values of doublings per day were calculated as previously described [24].

### Quantitative RT-PCR

Total RNA was isolated using the RNeasy Plus Mini Kit (Qiagen) according to the manufacturer’s instructions, and converted to cDNA using iScript cDNA Synthesis Kit (Biorad). Quantitative RT-PCR reactions were carried out on a LightCycler 480 II (Roche) using SYBR Green PCR Master Mix (Thermo Fisher Scientific). The 2^−ΔΔCt^ method was used to calculate fold change by using GAPDH as an internal control. The primers are listed in Additional file 2: Table S1.

### Immunoprecipitation and mass spectrometry

Whole cell lysates from triplicates per condition, each 5 × 10^6^ cells, were prepared using NP40 lysis buffer (Invitrogen, #FNN0021). EZview Red ANTI-FLAG M2 Affinity Gel (Sigma, #F2426) was added to each CTRL/CIC/CIC^S173A^ sample. For the binding step, the sample/bead mixtures were incubated overnight at 4 °C on a rotator and then washed with 1 ml of Wash Buffer for 10 min at 4 °C. The wash steps were repeated five times. IP samples were eluted with 3 × FLAG peptide. Proteomics were performed at the Mass Spectrometry Core Facility at the German Cancer Center (DKFZ).

### Glutamate measurement

The concentration of glutamate was measured using a colorimetric glutamate assay kit (Sigma, MAK004). Briefly, 20 µl of media samples were collected 3 days after cell seeding and mixed with 30 µl of glutamate assay buffer. The kit’s reaction mix was then added to each sample and incubated for 30 min at 37 °C. A glutamate-dependent color change at a wavelength of 450 nm was measured, and a standard curve was obtained using 0.1 M glutamate solution. Glutamate concentration was analyzed according to the manufacturer’s instructions.

### Harvest of TS603-conditioned media and PC12 cell viability assay

The determine the effect of glioma-derived conditioned media (CM) on PC12 cell viability, two million TS603 cells were plated in T75 flask and cultured in proliferation conditions for 3 days. The same culture media were incubated in T75 flask without cultivating cells and used as a control. Samples of culture media were filtered through 0.2 µm filter and kept at -20 until use. PC12 cells were cultured in 96-well plates until cells were approximately 70% confluent. The glioma-derived CM or control media were added to the experimental well in the 96-well plate. 72 h later, the cells were then added with 10 µl of MTT reagent (Trevigen) for 2 h, and solubilized with 100 µl of supplied detergent for 4 h at 37 °C. The cell viability was determined by measuring the absorbance at a wavelength of 570 nm, and the values were normalized using control media-treated cells.

### Bulk RNA sequencing and data analysis

RNA quantity and quality were measured using 4200 TapeStation (Agilent), and samples with an RNA integrity number greater than 9.8 were used for sequencing. RNA-seq libraries were generated with TruSeq Stranded mRNA Library Prep Kit (Illumina). Sequencing was carried out on the Illumina HiSeq 2000 using single-read 50 bp sequencing (DKFZ Genomics Core Facility). The data are available in the Gene Expression Omnibus. Quality of fastq files was validated with the software FastQC (www.bioinformatics.babraham.ac.uk/projects/fastqc). The STAR aligner was used to map the reads to a reference genome (hg19). hg-19-annotated genes were counted using FeatureCounts. Normalization of the raw counts and differential expression analysis were performed using DESeq2 package. GSEA was carried out with help of WebGestalt [17] (*q* < 0.05). Heatmap, Scatter and Volcano plots were generated using the pheatmap, ggplot2, ggrepel and EnhancedVolcano packages in R. *CIC* mutation status was identified from cBioPortal [1, 7]. *CIC* expression data were obtained from the GlioVis data portal [3].

### Statistical analysis

An unpaired Student’s *t*-test or one-way ANOVA was used to compare differences between two groups or among multiple experimental groups, respectively. Data are graphed as mean ± standard error of the mean (SEM) or standard deviation (SD). GraphPad Prism software version 8.3 was used for all significance calculation. P values below 0.05 were considered significant.

## Results

### Levels of CIC inversely correlate with its target ETV4 and cell proliferation of IDH1-mutant-codel gliomas

**Fig. 1:**
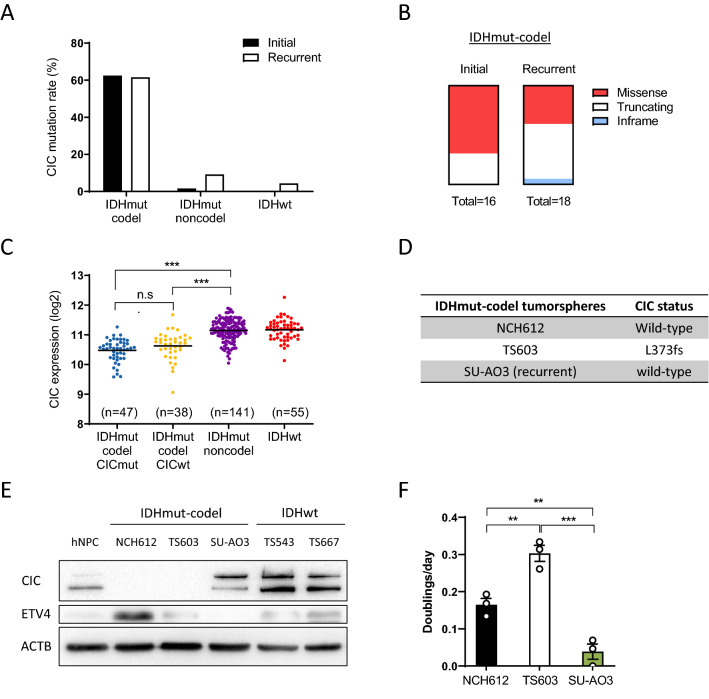
Identification of CIC alterations in IDHmut-codel gliomas. **A** CIC mutation rate stratified by each patient’s glioma subtype and tumor progression. **B** CIC mutation type in the initial and recurrent IDHmut-codel gliomas. **C** CIC expression in adult glioma subtypes (IDHmut codel CICmut, n = 47; IDHmut codel CICwt, n = 38; IDHmut non-codel, n = 141; IDHwt, n = 55). ***p < 0.001. **D** CIC mutation status in IDHmut-codel tumorspheres. **E** CIC and ETV4 protein levels in glioma tumorspheres and human neural progenitor cells (NPCs). **F** Doublings per day of IDHmut-codel tumorspheres. Mean ± SEM of at least three independent experiments. **p < 0.01, ***p < 0.001

CIC is frequently mutated in IDH-mutant and 1p/19q codeleted gliomas (referred to as IDHmut-codel) [6]. We examined the CIC mutation rates of the distinct molecular subtypes during tumor progression using cBioPortal [1, 7]. While CIC mutation rates in IDHmut-codel gliomas were comparable between the initial and recurrent stages of disease, IDHmut-noncodel and IDH wildtype gliomas exhibited a slight increase in mutation rate over time (Fig. 1A). Moreover, there is an increased occurrence of CIC truncating mutations in IDHmut-codel gliomas with disease progression (Fig. 1B).

To investigate whether CIC mutations affect its expression, we compared mRNA expression in TCGA glioma datasets. We detected a decrease in *CIC* expression in IDHmut-codel gliomas, associated with loss of heterozygosity of CIC due to chromosome 1p/19q loss (Fig. 1C). In contrast, no significant change in *CIC* expression was observed between CIC-mutant and CIC-wildtype IDHmut-codel subtypes (Fig. 1B). As previously reported, CIC mutations led to derepression of CIC target genes (*ETV1*, *ETV4*, and *ETV5*) (Additional file 1: Fig. S1). We next tested this finding using patient-derived IDHmut-codel tumorspheres [23]. Genetic profiling confirmed a CIC mutation (c.1118_1119insAC) in TS603 but not in NCH612 and SU-AO3 (Fig. 1D). As shown in Fig. 1E, CIC protein levels inversely correlated with ETV4 in human neural progenitor cells (NPC) and glioma tumorspheres. Consistent with this, loss of CIC was associated with faster-cycling cells in IDHmut-codel tumorspheres (Fig. 1F).

### Overexpression or knockout of CIC has no significant influence on cell proliferation and expression of its target genes in glioma tumorspheres

**Fig. 2:**
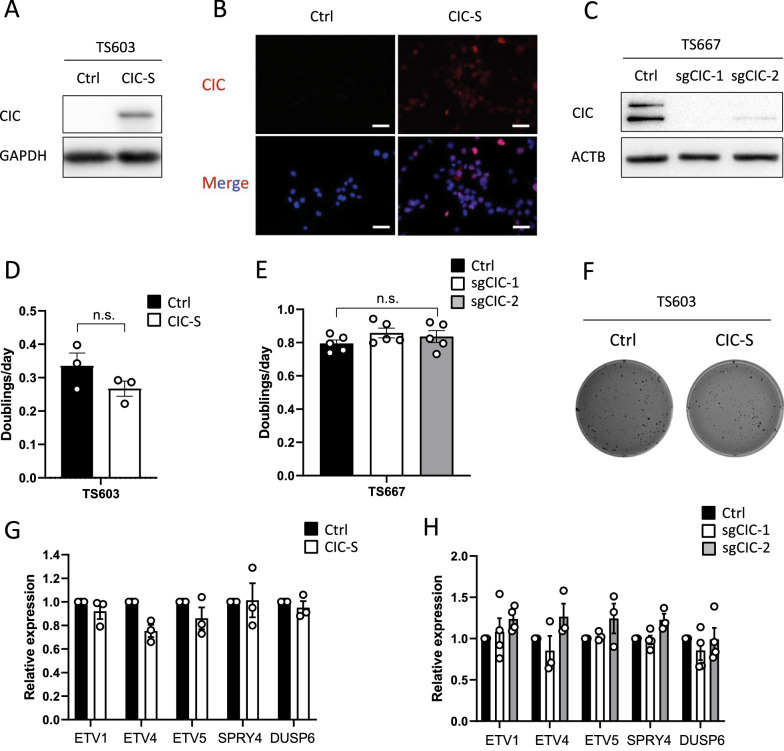
Effect of overexpression or knockout of CIC on glioma proliferation and its target genes. **A**, **B** TS603 tumorspheres were transduced with Ctrl or CIC as determined by immunoblot (**A**) and immunostaining (**B**) Scale bar = 40 μm. **C** TS667 tumorspheres were transduced with lentiCRISPR v2-puro (Ctrl) or lentiCRISPR v2-sgCIC1/2-puro, and CIC protein levels were assessed by immunoblot. **D**, **E** Cell proliferation rate of CIC-proficient and CIC-deficient glioma lines as assessed by MTT assay. **F** Colony formation of TS603 expressing Ctrl or CIC in soft agar. **G**, **H** The expression of CIC target genes in CIC-proficient and CIC-deficient glioma lines as determined by qPCR (n = 3). Mean ± SED of at least three independent experiments

To understand the biological consequences of CIC alterations in gliomas, we generated TS603 cells ectopically expressing full-length CIC protein (Fig. 2A). Mammalian cells express two CIC isoforms, CIC-S (short) and CIC-L (long). Since a subset of oligodendrogliomas harbors CIC-S-specific mutations but not L-specific mutations [28], we used CIC-S isoform for overexpression (OE) experiments. Consistent with its role as a transcriptional repressor, CIC proteins were predominantly localized in the nucleus (Fig. 2B). We also performed CRISPR/Cas9-mediated CIC knockout (KO) approaches to investigate the role of endogenous CIC in IDH-wildtype TS667 tumorspheres (Fig. 2C). As shown in Fig. 2D and E, the effect of CIC OE or KO on cell proliferation was modest, and no significant differences were found in TS603 and TS667 glioma tumorspheres. A similar trend was confirmed using the soft agar colony formation assay (Fig. 2F and Additional file 1: Fig. S2). Further supporting these findings, we tested whether CIC regulates the expression of its target genes *ETV1*, *ETV4*, *ETV5*, *SPRY4*, and *DUSP6*. Consistent with the above findings, there were no significant changes in the expression of CIC target genes (Fig. 2G and H), suggesting that the transcriptional repressor activity of CIC in gliomas may be blocked by mechanisms other than its mutations.

### Phosphorylation-insensitive form of CIC enhances its repressor activity and inhibits cell proliferation in TS603 line

**Fig. 3:**
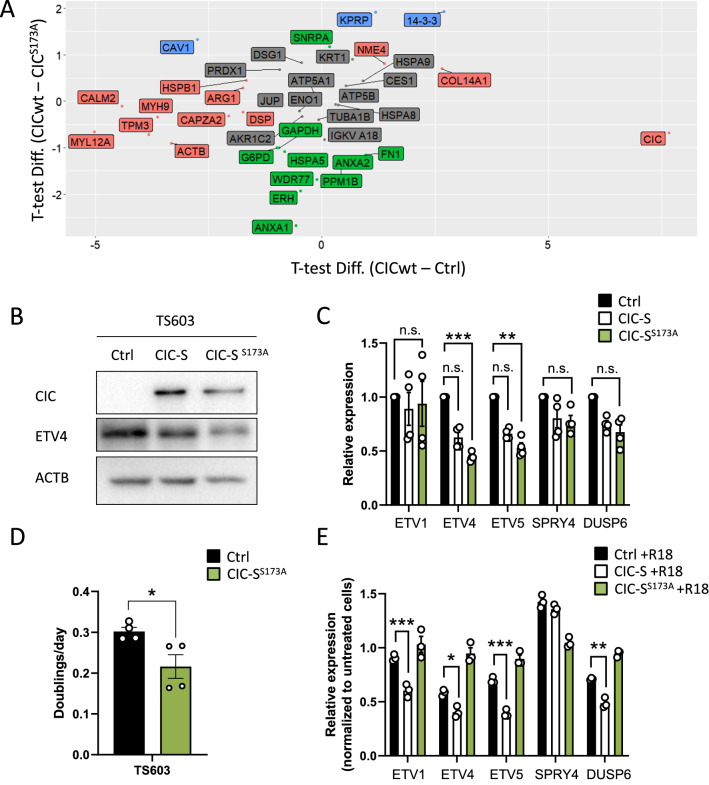
Effect of phosphorylation-insensitive form of CIC on its repressor activity and cell proliferation. **A** CIC interaction partners identified by mass-spectrometry of Flag immunoprecipitated TS603 cells ectopically expressing Flag-CIC or Flag-CIC (S173A). The indicated proteins with *t*T-test difference > 1 are highlighted in red (CIC-specific), green (S173A-specific), and blue (CIC^S173A^-specific). **B** ETV protein levels in TS603 cells transduced with Ctrl, CIC-S or CIC-S (S173A). **C** The mRNA expression of CIC targets in TS603 cells expressing Ctrl, CIC-S or CIC-S (S173A). **D** Cell proliferation rate of TS603 cells expressing Ctrl or CIC-S (S173A). **E** The expression of CIC target genes in R18 (14-3-3 inhibitor)-treated TS603 cells as determined by qPCR. The expression was normalized to values from untreated cells as a control. Mean ± SEM of at least three independent experiments. *p < 0.05, **p < 0.01, ***p < 0.001

CIC is negatively regulated by phosphorylation event on serine 173 residue [5, 10]. Moreover, numerous studies have shown that the phosphorylated S173 of CIC interacts with 14-3-3 proteins, reducing the DNA binding activity of CIC [10, 20, 32]. We validated the interaction between CIC and 14-3-3 in TS603 ectopically expressing Flag-CIC by performing Flag immunoprecipitation followed by mass spectrometry (IP-MS) (Fig. 3A and Additional file 3: Table S2). In addition to 14-3-3, we also detected known binding partners of CIC such as HSPA9, HSPA8 and ATP5B [9] (Additional file 1: Fig. S3).

To further explore phosphorylation-mediated CIC inactivation, we generated TS603 expressing CIC serine mutant (CIC^S173A^), in which serine 173 was replaced by alanine, using site directed mutagenesis (Fig. 3B). As expected, CIC^S173A^ binds with a low affinity to 14-3-3 compared to CIC wildtype (Fig. 3A). Interestingly, this phosphorylation-insensitive form of CIC (CIC^S173A^) enhanced the repressor activity on CIC target genes, particularly ETV4 and ETV5 (Fig. 3B and C). Furthermore, CIC^S173A^ OE significantly inhibited cell proliferation in TS603 line (Fig. 3D). Next, we assessed the effect of R18, 14-3-3 antagonist, on derepression of CIC’s target genes. R18-mediated transcriptional repression was more pronounced in TS603 expressing CIC wildtype but not the CIC serine mutant (CIC^S173A^) (Fig. 3E), suggesting that 14-3-3 inhibits the canonical function of CIC in TS603 through phosphorylated S173.

### Glutamate release is significantly increased in CIC-depleted human neural progenitor cells

**Fig. 4:**
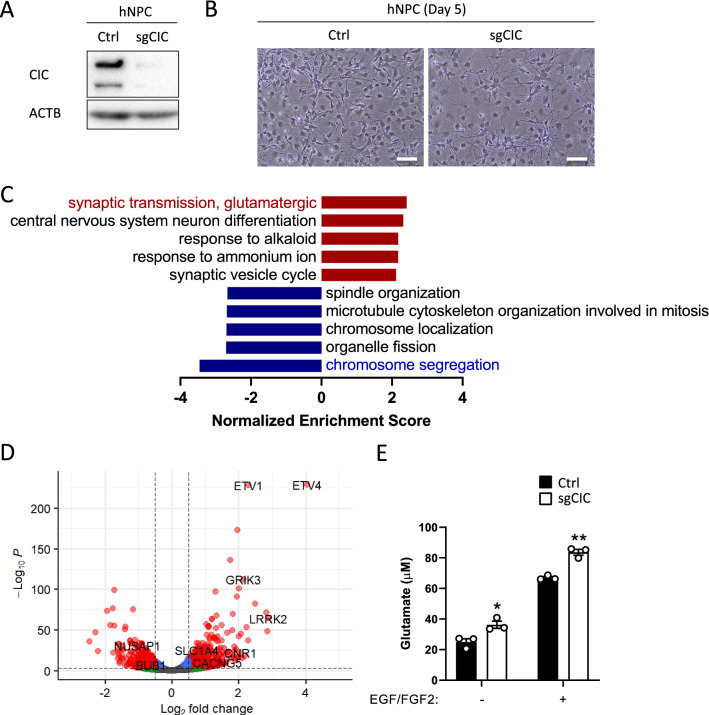
Transcriptional changes and glutamate secretion in CIC-depleted human neural progenitor cells. **A** Human neural progenitor cells (hNPCs) stably expressing Cas9 and sgCIC targeting CIC, as determined by immunoblot. **B** hNPC differentiation at day 5 after withdrawal of EGF and FGF2, scale bar = 40 μm. **C** Positively or negatively enriched top five GO terms in CIC-depleted hNPCs, as assessed by the normalized enrichment score of GSEA (*q* < 0.05). **D** Volcano plot showing DEGs between CIC-proficient and -deficient hNPCs. Red dots indicate significantly regulated DEGs. **E** Glutamate concentration in CIC-proficient and -deficient hNPCs with or without growth factors. Mean ± SEM of three independent experiments. *p < 0.05, **p < 0.01

The expression signatures of proliferating cells in oligodendrogliomas are closely associated with neural progenitor cells (NPCs) [30]. For this reason, we also utilized human NPCs for loss-of-function experiments to gain insight into CIC-mediated transcriptional changes (Fig. 4A). This cell line has been shown to differentiate into glial cells and neurons in culture when growth factors are removed [13]. As shown in Fig. 4B, hNPCs transduced with an empty lentiviral vector (Ctrl) or sgCIC displayed the differentiated phenotype in media without EGF and FGF2. To assess gene expression profiles, we performed RNA sequencing, and differentially expressed genes (DEGs) were selected based on q-value cutoffs (Additional file 4: Table S3). Upon CIC loss, 624 genes (61%) were upregulated, and 400 genes (39%) were downregulated (*q* < 0.05 and FC > 1.5). As expected, CIC deficiency resulted in increased expression of genes (*ETV1*, *ETV4*, and *ETV5*) (Additional file 4: Table S3). Interestingly, gene set enrichment analysis (GSEA) indicated that “chromosome segregation” is the most negatively enriched term in CIC-deficient hNPCs (Fig. 4C), confirming a recent finding from independent study (Takemon et al. 2022 preprint). In addition, depletion of CIC increased the expression of genes related to “glutamatergic synaptic transmission” (Fig. 4C). We found upregulation of genes (*LRRK2*, *GRIK3*, *SHC3*, *CACNG5*, *SLC17A8*, *SLC1A4*, *GRIA2*, and *CNR1*) involved in vesicular release of glutamate (Fig. 4D and Additional file 5: Table S4). To confirm these results, we measured the glutamate concentration in the culture media of both CIC-intact and CIC-deficient hNPCs. Consistent with the GSEA, increased levels of glutamate were observed in CIC-deficient hNPCs, regardless of the presence of growth factors (Fig. 4E).

### CIC overexpression or NMDA receptor antagonist abrogates glioma-conditioned media-mediated neuronal toxicity

**Fig. 5:**
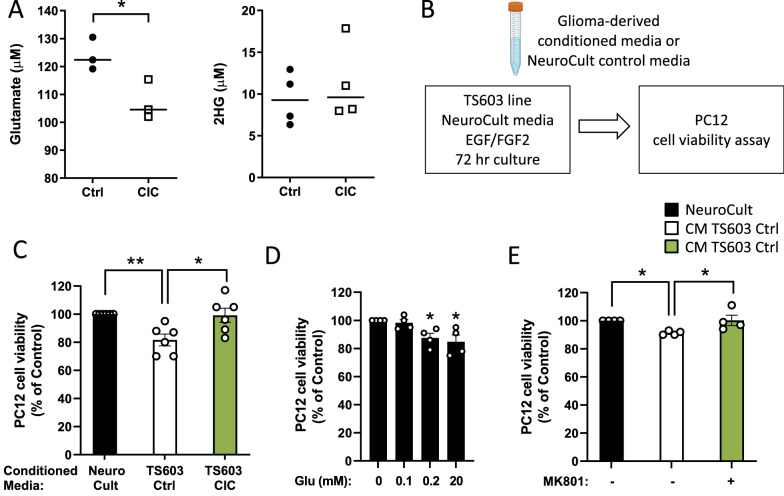
Effect of CIC overexpression or NMDA receptor antagonist on glioma-conditioned media-mediated neuronal toxicity. **A** Glutamate and 2-HG levels in culture media incubated with TS603 Ctrl or CIC line. **B** Schematic of conditioned media (CM) using TS603 lines in NeuroCult media for 72 h. The figure was created with BioRender.com. **C** PC12 cell viability in the CM harvested from TS603 cells transduced with Ctrl or CIC. **D** PC12 cell viability in response to glutamate treatment with the indicated concentrations. **E** PC12 cell viability in the CM with or without MK801 (NMDA receptor antagonist) as assessed by MTT assay. The absorbance value is normalized to value obtained from NeuroCult CM. Mean ± SEM of at least three independent experiments. *p < 0.05, **p < 0.01

To determine whether CIC regulates glutamate release in gliomas, we measured the concentration of glutamate in culture media collected from TS603 Ctrl and CIC OE lines. Interestingly, CIC OE significantly reduced glutamate secretion, but not 2-hydroxyglutarate (2-HG) levels, in the TS603 line (Fig. 5A). It has been reported that glioma cells release neurotoxic concentrations of glutamate, which ultimately lead to peritumoral neuronal hyperexcitability [4, 35]. To test whether CIC modulates neuronal toxicity, we cultured PC12 neuron-like cells in conditioned media (CM) harvested from the TS603 line (Fig. 5B). As shown in Fig. 5C, the CM from TS603 Ctrl line inhibited the viability of PC12 cells, while CIC OE rescued the impaired PC12 cell viability. In line with this, treatment of PC12 cells with glutamate attenuated cell viability in a dose-dependent manner (Fig. 5D). Furthermore, treatment with 10 µM of MK801 (N-methyl-d-aspartate receptor antagonists) prevented the reduced PC12 cell viability, suggesting that the glioma-induced loss of cell fitness in PC12 may be due to the hyperactivation of glutamate receptors (Fig. 5E).

### SLC7A11/xCT expression and its protein levels are significantly reduced in CIC OE TS603

**Fig. 6:**
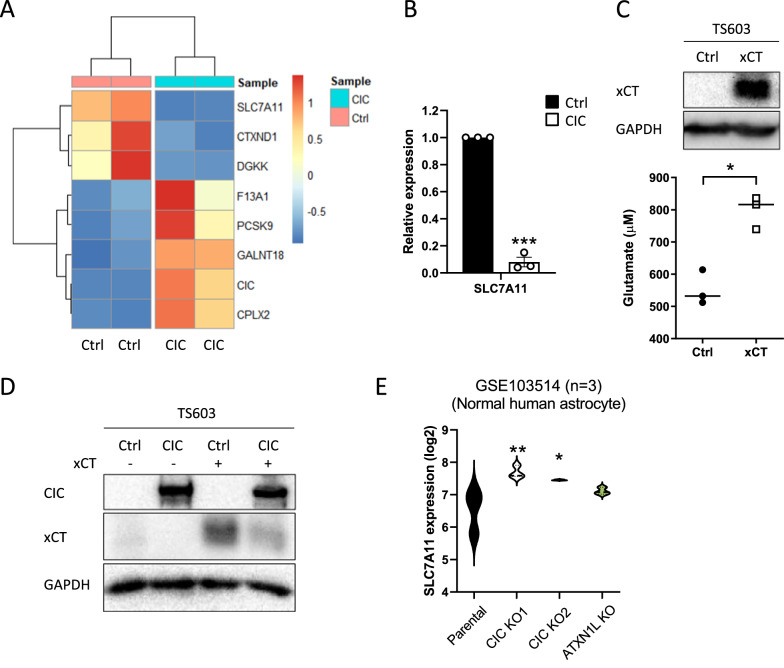
*SLC7A11*/xCT expression and its protein levels are significantly reduced in CIC OE TS603. **A** Differential gene expression analysis of RNA-seq in TS603 Ctrl and CIC cells. **B** SLC7A11 mRNA expression in TS603 Ctrl and CIC cells as validated by RT-qPCR. **C** Glutamate levels (lower) in TS603 ectopically expressing Ctrl or xCT (upper). **D** SLC7A11/xCT protein levels in TS603 Ctrl and CIC cells transduced with xCT. (E) SLC7A11 mRNA expression in the indicated NHA derivatives from GSE103514 dataset. Mean ± SEM of at least three independent experiments. *p < 0.05, **p < 0.01, ***p < 0.001

Previous studies have shown that the cystine/glutamate antiporter xCT (SLC7A11) is responsible for the glutamate release pathway in gliomas [4, 25]. Strikingly, RNA-sequencing analysis revealed the reduced *SLC7A11* expression in TS603 CIC OE cells (Fig. 6A), which was also validated by RT-qPCR (Fig. 6B). Consistent with its role as a cystine/glutamate transporter, xCT overexpression enhanced glutamate release in TS603 cells (Fig. 6C). In accordance with the reduced expression of SLC7A11 mRNA, CIC overexpression decreased the levels of xCT protein, particularly in cells ectopically expressing xCT (Fig. 6D). Analysis of publicly available RNA-seq data [33] obtained from normal human astrocyte (NHA) cells with loss of CIC and ATXN1L showed significantly elevated *SLC7A11* expression in two independent CIC- but not ATXN1L-deficient conditions (Fig. 6E), indicating that CIC-mediated SLC7A11 modulation is not a TS603-specific event. Given that ETS1 directly transactivates the xCT promoter [18], we queried TCGA dataset and identified an inverse correlation between *CIC* and *ETS1* expression, particularly in the IDHmut-codel subtype (Additional file 1: Fig. S4). Moreover, we found that significant downregulation of *ETS1* occurs in TS603 cells overexpressing CIC (Additional file 6: Table S5). Taken together, these results demonstrate that CIC regulates glutamate release in glioma cells possibly via SLC7A11.

## Discussion

CIC mutations correlate with a more malignant subtype in a variety of cancers, including ODGs [8, 12]. We found that IDHmut-codel gliomas exhibit increased truncating CIC mutations during tumor progression (Fig. 1B), indicating a strong selective pressure on clones with more aggressive clinical phenotypes [27].

In addition to the genetic alterations, CIC inactivation can also be mediated by post-translational mechanisms. For instance, the E3 ligase PRAJA1 (PJA1) is highly expressed in GBM and PJA1 induces CIC degradation [5]. Similarly, CIC proteins are not detected in CIC wildtype NCH612 (Fig. 1E), suggesting a rapid degradation of CIC in the IDHmut-codel gliomas [8]. Given that CIC inactivation could be mediated by genetic mutations or post-translational mechanisms, CIC restoration needs to be explored in ODG.

ETV1/4/5, the most well-known targets of CIC, are associated with cell proliferation, invasion, and treatment resistance. Our study demonstrated that CIC restoration alone is not sufficient for its repressor activity on the ETS factors in ODG. Similarly, other studies showed that CIC reconstitution does not bring drug sensitivity in CIC-deficient cancer cells [16, 21, 31]. In our reconstitution setting, transcriptional repression of CIC becomes functional when 14﻿-3-3 is inactivated by the pharmacological inhibition (Fig. 3E). Supporting this, the CIC serine mutant (CIC^S173A^), which is unable to interact with 14-3-3, significantly reduces the expression of target genes and cell proliferation in the TS603 line (Fig. 3D).

The function of CIC independent of RTK/MAPK was explored in CIC conditional knockout mice [34]. Our data reveal that ectopic CIC expression reduced the levels of xCT (SLC7A11) and glutamate release in glioma cells. Decreased expression of *SLC7A11* is also observed in TS603 expressing CIC^S173A^ (data not shown). We further confirmed that CIC overexpression rescues the impaired PC12 cell viability from glioma-derived culture media. In parallel with these findings, the treatment of MK801, an antagonist of the N-Methyl-D-aspartate (NMDA) receptor, blocks the impaired viability in PC12 cells exposed to the conditioned media, demonstrating that the toxic effects are dependent on the glutamate channel.

We validated the relationship between CIC and xCT (SLC7A11) in transcriptomic analysis (GSE103514) from an independent study using the NHA cell line [33]. Interestingly, in human NPC culture without EGF and FGF2 treatment, *SLC7A11* is not significantly upregulated by loss of CIC. This discrepancy may be due to heterogeneously differentiated conditions resulting in multiple cell populations such as neurons and glial cells. Instead, other genes related with vesicular release of glutamate are upregulated in CIC-deficient hNPCs.

Importantly, our results demonstrate that CIC regulates glutamate secretion in CIC-altered NPC and glioma cells, although further research is needed to investigate the underlying mechanism by which xCT expression is modulated by CIC. Taken together, our findings provide insight into glioma-related neuronal hyperexcitability in IDH-mutant gliomas.

## Supplementary Information

**Additional file 1: Figure S1.** The expression of PEA3/ETS transcription factors in CIC-mutant gliomas. **Figure S2.** Soft agar colony formation assay of TS667 cell derivatives. **Figure S3.** CIC protein interaction networks from STRING. **Figure S4.** ETS1 and CIC expression in TCGA adult glioma subtypes.

**Additional file 2: Table S1**. List of aRT-PCR primers used in this study.

**Additional file 3: Table S2**. CIC binding partners identified by mass-spectrometry

**Additional file 4: Table S3**. Differentially regulated genes with q < 0.05 in CIC-deficient hNPCs.

**Additional file 5: Table S4**. Enriched gene sets in CIC-deficient hNPCs.

**Additional file 6: Table S5**. Differentially regulated genes with q < 0.01 and FC > 1.5 in CIC-OE TS603.

## Data Availability

All data will be made available upon request.
