# Supplementary material for: CIC reduces xCT/SLC7A11 expression and glutamate release in glioma
Source: Acta Neuropathol Commun. 2023 Jan 16;11:13. doi: 10.1186/s40478-023-01507-y (PMC9843885; doi:10.1186/s40478-023-01507-y)
Supplement: Supplementary file 1 — Additional file 1: Figure S1. The expression of PEA3/ETS transcription factors in CIC-mutant gliomas. Figure S2. Soft agar colony formation assay of TS667 cell derivatives. Figure S3. CIC protein interaction networks from STRING. Figure S4. ETS1 and CIC expression in TCGA adult glioma subtypes. [file 40478_2023_1507_MOESM1_ESM.docx]

**CIC reduces xCT/SLC7A11 expression and glutamate release in glioma**

Jong-Whi Park^1,2*^, Omer Kilic^1^, Minh Deo^1^, Kevin Jimenez-Cowell^1^, Engin Demirdizen^1^, Hyunggee Kim^3^, Şevin Turcan^1*^

^1^Neurology Clinic and National Center for Tumor Diseases, University Hospital Heidelberg, INF 460, Heidelberg, 69120, Germany.

^2^Department of Life Sciences, Gachon University, Incheon, 21999, South Korea

^3^Department of Biotechnology, College of Life Sciences and Biotechnology, Korea University, Seoul, 02841, South Korea

**Corresponding Authors**: Şevin Turcan, Phone: +49-(0)6221-56-5929, Fax: +49-(0)6221-56-5978, (sevin.turcan@med.uni-heidelberg.de). Jong-Whi Park, Phone: +82-(0)32-899-6115, Fax: +82-(0)32-899-6039, (jpark@gachon.ac.kr).
